# Disrupted visual input unveils the computational details of artificial neural networks for face perception

**DOI:** 10.3389/fncom.2022.1054421

**Published:** 2022-11-29

**Authors:** Yi-Fan Li, Haojiang Ying

**Affiliations:** Department of Psychology, Soochow University, Suzhou, China

**Keywords:** artificial neural networks (ANN), face perception, cognitive science, vision, computational vision

## Abstract

**Background:**

Convolutional Neural Network (DCNN), with its great performance, has attracted attention of researchers from many disciplines. The studies of the DCNN and that of biological neural systems have inspired each other reciprocally. The brain-inspired neural networks not only achieve great performance but also serve as a computational model of biological neural systems.

**Methods:**

Here in this study, we trained and tested several typical DCNNs (AlexNet, VGG11, VGG13, VGG16, DenseNet, MobileNet, and EfficientNet) with a face ethnicity categorization task for experiment 1, and an emotion categorization task for experiment 2. We measured the performance of DCNNs by testing them with original and lossy visual inputs (various kinds of image occlusion) and compared their performance with human participants. Moreover, the class activation map (CAM) method allowed us to visualize the foci of the “attention” of these DCNNs.

**Results:**

The results suggested that the VGG13 performed the best: Its performance closely resembled human participants in terms of psychophysics measurements, it utilized similar areas of visual inputs as humans, and it had the most consistent performance with inputs having various kinds of impairments.

**Discussion:**

In general, we examined the processing mechanism of DCNNs using a new paradigm and found that VGG13 might be the most human-like DCNN in this task. This study also highlighted a possible paradigm to study and develop DCNNs using human perception as a benchmark.

## 1. Introduction

Deep Convolutional Neural Network (DCNN) is a type of Artificial Neural Network (ANN) that was largely inspired by Hubel and Wiesel's research on the human visual neural system (Hubel and Wiesel, 1959, 1962). After decades of iteration, researchers now have built several DCNNs that can perform on par with humans on many computationally complicated tasks. AlexNet (Krizhevsky et al., 2012) is the first deep neural network proposed, which introduced the concept of depth to neural networks for the first time in practice based on the traditional convolutional neural network LeNet (LeCun et al., 1998), and caused the birth of deep learning as a machine learning subfield and brought it to widespread attention. In the follow-up study, the network continues to deepen, from VGGNet (Simonyan and Zisserman, 2014), which can reach up to 19 layers, as proposed in 2014, to ResNet (He et al., 2016), which can achieve hundreds of layers. In addition to these classical DCNNs, some widely popular DCNNs have been developed in recent years through improved algorithms, such as DenseNet (Huang et al., 2017), which densely connects different convolutional layers, MobileNet (Howard et al., 2017, 2019; Sandler et al., 2018), which improves the convolutional method to reduce the parameters and training difficulty and maintain the performance, and EfficientNet (Tan and Le, 2019, 2021), which improves on MobileNet by introducing three hyperparameters: input resolution factor, network width and depth factor, and introduces dynamic learning method, etc. the deep neural network DCNN has the accuracy and performance to reach or even surpass human in tasks such as target detection, semantic segmentation, image classification, etc (Weyand et al., 2016; Yu et al., 2017; Larmuseau et al., 2021). The performance of DCNN has gradually improved over the years, not only for specific tasks such as face-based identification tasks but also for general tasks such as visual perception of liquids (van Assen et al., 2020), shape perception (Kubilius et al., 2016), scene segmentation problems (Seijdel et al., 2020), etc.

DCNN, typically with millions of parameters, is widely regarded as a "black box", which means that researchers currently do not have a thorough understanding of its specific mechanisms and principles. Although, it is well known that neural network models are inspired by the visual neural system (Hubel and Wiesel, 1959, 1962); however, certain mechanisms used in DCNN such as error back propagation (BP) learning methods are considered to be impossible to occur in biological neural networks by neuroscientists (Lillicrap et al., 2020). Some other artificial intelligence methods such as vision transformer (ViT) (Dosovitskiy et al., 2020) that have recently achieved good performance seem to have mechanisms of action that are more distant from the biological neural system. However, recent advancements in artificial neural network studies highlighted the importance of neural network structures but less on the functional and computational mechanisms. Some recent studies indicated that a renewed focus on the inspiration of the human nervous system for neural networks could be more helpful for the further improvement and development of artificial intelligence. For example, the deep network PredNet (Lotter et al., 2016) and the neural network robustness enhancement package Predify (Choksi et al., 2021) are inspired by the concept of predictive coding observed in neuroscience. In addition, the brain-inspired replay method effectively solves the problem of catastrophic forgetting of artificial neural networks (van de Ven et al., 2020). And the recently re-emphasized spiking neural network introduces a time series model of biological neurons (Ghosh-Dastidar and Adeli, 2009; Tavanaei et al., 2019).

Reciprocally, having brain-inspired DCNN allows researchers to use neural networks as a computational model for the study of the human neural system. In some recent studies, DCNN has been commonly considered to be studied as a framework of the human visual system (Kriegeskorte, 2015; Kietzmann et al., 2018; Yang and Wang, 2020). For the exploration of single neurons in artificial neural networks, receptive field analysis (Mahendran and Vedaldi, 2015; Yosinski et al., 2015) has shown that artificial neurons have similar performance to ventral pathways (Luo et al., 2016), and experiments related to ablation analysis (Morcos et al., 2018; Zhou et al., 2018) have tentatively confirmed the possibility of ablation analysis of single neurons in theoretical neuroscience (Barrett et al., 2016), which is difficult to be performed in biological neural networks (Miller et al., 1991). Additionally, some recent studies have also confirmed that CNN models also have some similarities with humans in gender classification tasks by means of reversing correlation in terms of information acquisition (Song et al., 2021). In another recent study, passive attention techniques also revealed a significant overlap between the selective estimation of artificial neural networks and that of human observers (Langlois et al., 2021). In typical neural network evaluation criteria, the performance of other neural networks is generally used as the baseline. The layer-by-layer convolutional structure of DCNN can simulate the human visual system in a better way, so it is possible to evaluate the performance of neural networks using humans. Here, we not only tested the external behavior of DCNNs with human psychophysics data, but also studied the information extraction pattern of the DCNNs to further examine their processing mechanisms. This paradigm would utilize the understanding of human performance from cognitive science, as well as the architecture of the human neural systems and DCNNs, to offer a better understanding of DCNNs from the perspective of function and processing mechanisms.

In this study, we further tested and explored the performance and interpretability of the neural network by using human performance as a benchmark for neural networks. We tested seven deep convolutional neural networks, AlexNet, VGG11, VGG13, VGG16, DenseNet, MobileNet, and EfficientNet on a simple ethnicity categorization task.

## 2. Methods

### 2.1. Overall experimental procedure

This study consisted of several parts of the experiment. At first, we measured the human performance (psychometric function) and set them as a benchmark to unveil the mechanisms of DCNNs. If a DCNN utilizes the processing mechanism as humans, then it shall behave the same (in terms of psychometric function) as humans. Moreover, from a backward correlation perspective, if the DCNN is indeed human-like, then it shall use the same visual input from faces (note that, each face offers abundant visual information). Thus, certain input impairments that would not affect human perception shall not affect human-like DCNNs as well. On the other hand, the human-like DCNNs shall 'attend' to the same regions that humans do.

Here in this study, we tested the DCNNs with different kinds of impaired inputs. In Experiment part 1, we compared the performance of humans and DCNNs with gray-scaled inputs. In Experiment part 2, we compared the DCNNs with essential facial features masked. And in Experiment part 3, we further studied their performance with only essential facial features available. To further interpret the results, we took a modified Grad-CAM-based LayerCAM neural network attention visualization approach for each part of the experiment.

### 2.2. Artificial neural networks

#### 2.2.1. Neural network architectures

In this study, we utilized 7 directly trained DCNNs in ethnicity categorization task. The AlexNet is an 8-layer deep convolutional neural network, which consists of five convolutional layers and three fully connected (FC) layers, where the convolutional layers are used for feature extraction calculation of the image. The FC layers are used for the final weighted classification. Nonlinear activation of Rectified Linear Unit (ReLU) (Glorot et al., 2011) is performed after each layer (including convolutional and FC layers, except the last one) to prevent gradient explosion or disappearance. After the convolutional layers of 1, 2, and 5 layers, a MaxPool operation is taken to compress the features and information to alleviate the over-sensitivity of the convolutional layers to the position. The Dropout (*p* = 0.5) (Srivastava et al., 2014) operation is also used before the first two fully connected layers to discard some of the neural links to reduce the possibility and degree of overfitting. Because we used single GPU training and thus the actual parameters passed in the convolutional layers would not match those reported in the original paper, we used a different set of parameters that the original authors subsequently matched for single GPU training (Krizhevsky, 2014). The VGGNets are overall deeper than the AlexNet with a smaller convolutional kernel operation, and use a structure called VGG blocks. Usually, a VGGNet contains five VGG blocks and three FC layers, therefore its general structure is similar to that of AlexNet. VGG11, VGG13, and VGG16 represent the 11-layer 13-layer 16-layer VGG network (Simonyan and Zisserman, 2014), respectively, also using ReLU nonlinear activation after each layer except the last one, and using MaxPool operation after each VGG block, and using dropout method in the first two fully connected layers. DenseNet (Huang et al., 2017) is a kind of ANN based on the development of the ResNet, and the DenseBlock is utilized by connecting all the convolutional layers through residuals, and preventing the generation of excessively high-dimensional feature matrices by transition layer. In our experiments, we utilized and tested DenseNet-201. MobileNet (Howard et al., 2017) uses depthwise separable convolution, which decomposes the normal convolutional layers into Depthwise (DW) convolution and Pointwise (PW) convolution, where DW convolution uses a three-channel convolutional kernel to process the input, and PW convolution uses multiple 1×1 convolutional layers. The PW convolution uses multiple 1×1 convolution kernels to up-dimension the result of DW convolution, replacing the normal convolution operation with two smaller convolution operations, which can significantly reduce the parameters of the network and reduce the computing pressure. In its V2 version (Sandler et al., 2018), the Inverted Residual block is introduced to take advantage of the better properties of DW convolution for high-dimensional data processing, and the information loss in convolutional operations is reduced by first up-dimensioning and then down-dimensioning, while in its V3 version (Howard et al., 2019) the Squeeze-and-Excitation (SE) channel attention module is introduced on the basis of the inverted residual block, which can obtain better performance by increasing a small amount of computation. In our experiments, we utilized and tested MobileNetV3-Large. EfficienitNet (Tan and Le, 2019) introduces three hyperparameters to adjust the network structure, i.e., increasing the depth, width, and input resolution of the network, which can further improve the performance of the network by mixing these three parameters, and the main body of the network structure adopts the deconvolution layer of MobileNet, which can balance the reduction of the network structure with the deepening of the network structure. parameters. In its V2 version (Tan and Le, 2021), the DW and PW convolutions are merged back into a normal 3×3 convolution in the early convolution operation, and MBConv is still used in the later layers. progressive learning is used to dynamically adjust the size of the input and the regularization method during training. In our experiments, we utilized and tested EfficientNetV3-S. We used the open-source PyTorch (Paszke et al., 2019) deep learning framework to build our neural network and used its default kaiming_normal method for initialization.

#### 2.2.2. Device and environment

The network training and image pre-processing were performed on a computer running Windows 10 (Microsoft, WA) OS with AMD Ryzen 1700 CPU, 16GB ram, and a GTX1070 GPU. The neural network framework was built using PyTorch, using compute unified device architecture (CUDA) version 11.6, PyTorch version 1.11.0, and the code was implemented in Anaconda environment with python environment (3.9.7).

#### 2.2.3. Dataset processing

The VGGFace2 Mivia Ethnicity Recognition (VMER) dataset (Greco et al., 2020) based on VGGFace2 was used to train the neural networks for ethnicity categorization. This dataset has 3M images containing 9,131 individuals. We choose faces from East Asian and Caucasian ethnicities for the study, and in order to balance the training time with the training effect, we selected images equally from each ethnic group in the dataset, which not only balances the differences between individuals, but also balanced the numbers of faces from the two ethnicities in the final dataset, and does the same for the training and test sets. We did this for the reason that recent studies have shown that unequal data sets can lead to bias in the neural network (Tian et al., 2021). Finally, we created a training set containing 10,718 Caucasian and 10,660 East Asian and a validation set containing 1,140 Caucasian and 1,240 East Asian for the neural network to learn.

To better validate the DCNNs, and to conduct a direct comparison between humans and DCNNs, we prepared an additional test set, which is a stream of 21 typical Caucasian male faces and a typical Eastern Asian male face deformed between the two types of Caucasian faces from London Face dataset (DeBruine and Jones, 2017) and Eastern Asian faces from the dataset (Yu and Ying, 2021). These faces are carefully aligned and masked with only inner features visible. Then, we controlled and equalized the luminance of these images using SHINE toolbox (Willenbockel et al., 2010).

#### 2.2.4. Neural network training

For the seven neural networks, based on our past experimental experience, we used the same settings, set batchsize to 32 (except for EfficientNet which was set to 16 due to hardware [more specifically, vram] limitation) and learning rate to 5 × 10^−5^, directly trained each network for 60 epochs in the dataset we extracted from VMER for ethnicity categorization task (Caucasian or Asian), and saved the model parameter data with the highest accuracy on the validation set as the model for the following experiments. Before the actual training, we also performed random clipping of the training set and random horizontal flipping to enhance our dataset.

### 2.3. Human experiment

The human data were from an existing experiment testing the same perceptual task. We reused the data as a benchmark to test the performance of the DCNNs.

#### 2.3.1. Participant information

Thirty human participants (mean age = 20.3; 21 females and 9 males) volunteered for the ethnicity perception experiment (part of data from a study currently under review). They offered written consent before the experiment. This study was approved by the Ethics Committee of Soochow University.

#### 2.3.2. Experiment procedure

The procedure of this experiment is adapted from a recent cognitive study using psychophysics methods (Ying et al., 2020).

In general, participants were asked to judge the perceived ethnicity of the testing faces (one at a time) *via* a Two-Alternative-Forced-Choice (Asian or Caucasian) paradigm by pressing A (Asian) or S (Caucasian) on the keyboard. Every participant completed 420 trials (21 testing faces ×20 repetitions) with randomized orders.

The testing stimuli are 21 faces genderated in a similar way as Ying et al. (2020). They were created by morphing between a typical Caucasian male face and a typical Eastern Asian male face using WebMorph software. The two typical faces are the averaged faces of Caucasian male faces from the London Face dataset (DeBruine and Jones, 2017) and Eastern Asian face was the average of all Asian male Faces from the dataset (Yap et al., 2016). These faces are carefully aligned (by the two eyes) and masked with only inner features visible. At last, the luminance of the images was controlled and equalized using the SHINE toolbox (Willenbockel et al., 2010).

The human behavioral experiment was conducted on a PC running Matlab R2016a (MathWorks) *via* PsychToolBox extensions (Brainard and Vision, 1997; Pelli and Vision, 1997) with a 27-inch LCD monitor (spatial resolution 1, 920 × 1, 080 pixels, refresh rate 120 Hz. During the experiment, participants sat in an adjustable chair, with their chins resting on a chin rest which was placed at 85*cm* away from the monitor, and each pixel subtended 0.025 on the screen.

#### 2.3.3. Comparison using psychophysics methods

The results of the psychophysical experiment of human participants were used as the baseline to evaluate the performance of the neural network at other variable levels. Psychophysical experimental methods were adapted from previous psychological studies (Webster et al., 2004) that asked each subject to judge the ethnic classification of the face tested from two options [press A (Asian) or S or (Caucasian) on the keyboard to make a judgment from two options.]. Each subject performed 420 trials, (21 faces × 20 repetitions), in a randomized order. For the machine, the same 21 face images were given to the machine for recognition, and after a complex CNN operation, a Softmax function was used for the resultant values to calculate their recognition probability for the resulting faces. Based on previous studies in Webster et al. (2004), different people with different standards of ethnicity judgments shall have different points of subjective equality (PSE). The PSE of each curve was then measured. With the above data, we can derive the PSE for humans and DCNNs, and we can then compare the PSE of the two types of curves. We used the *t*-test to compare the performance of each DCNN against the PSEs of 30 participants (the PSE of each DCNN against the mean and deviation of the human participants). Based on this, we will also submit the masked face images to DCNNs for the same operation and also baseline with the above human data to go further with our experimental study.

### 2.4. Attention area analysis of neural networks and human

To further validate our hypothesis on the similarities and differences between neural networks and humans about information processing, we employed different neural network interpretability methods. The majority of neural network interpretability methods can be divided into four types, visual interpretation methods, interference-based interpretation methods, knowledge-based interpretation methods, and causal interpretation methods. The most intuitive approach is the visual interpretation approach (Sun et al., 2022). Consequently, we chose to compare neural networks with humans from the perspective of attention. For humans, eye-tracking (Holmqvist et al., 2011; Duchowski, 2017) methods are a common method to study visual attention. The gaze and fixation of eye movements of human observers can be analyzed by devices such as eye-tracker to be able to derive human attentional tendencies (Blais et al., 2008; Miellet et al., 2013; Brielmann et al., 2014; Hu et al., 2014; Arizpe et al., 2017), and the results are generally derived by plotting heat maps. The results are often demonstrated in the form of a semi-transparent heat map overlaid on the original input image. For neural networks, the most widely used method for visual interpretation analysis is the class activation map (CAM) (Zhou et al., 2016), and a series of other improved methods. CAM replaces the fully connected layer of the original network for classification with global average pooling (GAP) and uses the GAP operation on each channel of the feature layer extracted from the convolutional layer to obtain the weights and perform a simple weighted summation with the feature layer channels to obtain the activation map of the neural network, which is expressed mathematically as follows.


(1) $$\begin{array}{l} M_{\left(x,y\right)}^{c}=\underset{k}{\sum }\left(\frac{1}{W\times H}\underset{x}{\sum }\underset{y}{\sum }A_{xy}^{k}\right)A_{xy}^{k} \end{array}$$

This method, as early works on visual interpretation of neural networks, enables us to obtain the region of interest of the neural network, but still has some shortcomings. It is modified directly on the original network and needs to be retrained before the results can be obtained, which will inevitably have a negative impact on the results. In subsequent work, the Grad-CAM (Selvaraju et al., 2017) solved this problem by back-propagating the gradient to obtain the desired weights equivalently and introducing the ReLU activation function to remove the result of negative activation, which is finally represented as follows.


(2) $$\begin{array}{l} M_{\left(x,y\right)}^{c}=ReLU\left(\underset{k}{\sum }\left(\frac{1}{W\times H}\underset{x}{\sum }\underset{y}{\sum }\frac{\partial y^{c}}{\partial A_{xy}^{k}}\right)A_{xy}^{k}\right) \end{array}$$

Grad-CAM methods are currently the most widely used methods for this type of question, while studies using gradient-based CAM methods combined with eye-movement analysis are still relatively rare, and we believe this is an area worth developing, and some researchers are currently trying and following up on this, for example, in this study (Alarifi et al., 2019) comparing eye-tracking experiments with activation maps for age estimation, and in another recent study (Ralekar et al., 2021), the authors also compared eye-tracking data with neural network attention regions for character recognition, combining eye-tracking analysis with CAM methods in this paper (Langlois et al., 2021). It is important to note that there are still few studies that combine the two. The above-mentioned studies, all pointed to the conclusion that humans and neural networks have some similarities in information acquisition and processing, which is a very interesting finding, and our paper also aims to conduct some experimental exploration of this relatively empty field through a similar approach in this way. In our specific study, we intend to use the newer LayerCAM (Jiang et al., 2021) method proposed by Jiang et al. based on a modified version of Grad-CAM.


(3) $$\begin{array}{l} M_{\left(x,y\right)}^{c}=ReLU\left(\underset{k}{\sum }\left(ReLU\frac{\partial y^{c}}{\partial A_{xy}^{k}}\right)A_{xy}^{k}\right) \end{array}$$

Note that, both the Grad-CAM and the LayerCAM are based on gradient backpropagation. However, the LayerCAM utilizes element-level weights; thus, it offers a more refined performance than Grad-CAM.

## 3. The experiment

### 3.1. Experiment part 1: Comparing the performance with grayscale and color inputs

Humans can correctly and effortlessly categorize the ethnicity of a grayscaled face from its shape, as many cognitive science studies used grayscale faces to test face perception. Therefore, a human-like DCNN shall perform the same as humans in terms of the psychometric function with or without color information. In this experiment, we exploit the neural network layer-by-layer attention area by CAM in five stages, with better performance in the earlier layers, which is the advantage of our choice of LayerCAM.

#### 3.1.1. Results of experiment part 1

**Figure 1:**
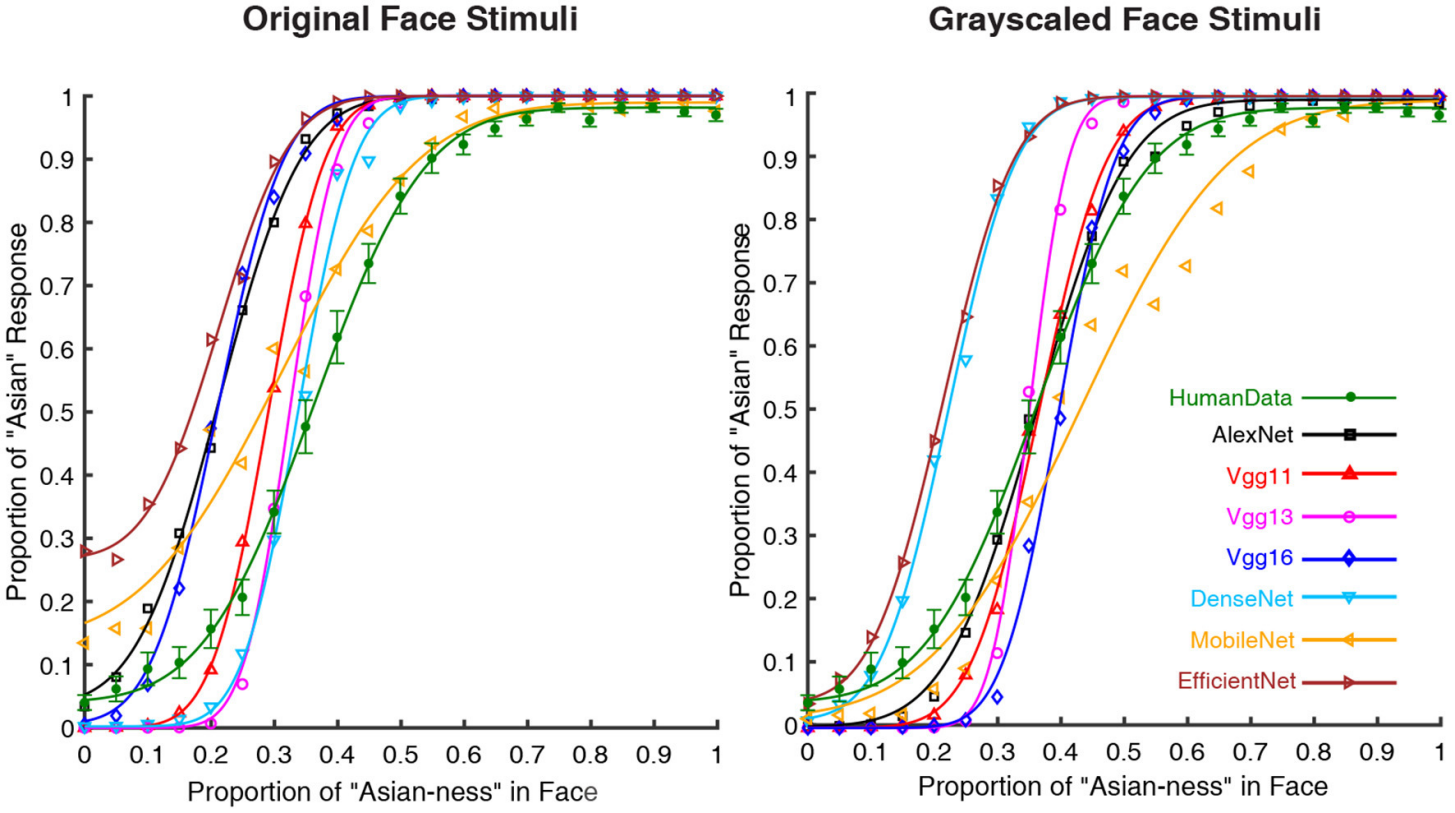
Psychometric functions of human participants and DCNNs under grayscale and color input conditions in Experiment Part 1. Green lines and symbols represent human participants, black lines and symbols represent AlexNet, red lines and symbols represent VGG11, magenta lines and symbols represent VGG13, blue lines and symbols represent VGG16, azure lines and symbols represent DenseNet, yellow lines and symbols represent MobileNet and brown lines and symbols represent EfficientNet. The horizontal coordinates represent the change in “Asian-ness” of the input, and the vertical coordinates represent the transformed predicted probabilities corresponding to human subjects or DCNNs.

The results of the psychometric functions were shown in Figure 1. It was obvious that the performance of human participants (green lines) followed a psychometric function (*M* = 0.362, *SEM* = 0.012). The seven DCNNs also followed similar performance as human participants based on the psychophysical curves. The statistical results of PSE (point of subjective equality) were shown in Table 1 (the *p*-values were Bonferroni corrected), For the grayscaled input condition, the performance of AlexNext, VGG11, and VGG13 closely resembled the performance of human participants in terms of PSE (all *p*-values closed to 1), the performance of VGG16 [*t*_(29)_ = 2.971, *p* = 0.083] is not significantly different from humans. However, the performances of much deeper ANNs significantly deviate from human participants: DenseNet [*t*_(29)_ = –11.826, *p* < 0.001], MobileNet [*t*_(29)_ = –5.858, *p* < 0.001], and EfficientNet [*t*_(29)_ = –12.540, *p* < 0.001]. On the contrary, for the color input condition, all DCNNs performed differently (all *p* < 0.005) from human participants apart from the DenseNet [*t*_(29)_ = –2.038, *p* = 0.711] (Table 1). Therefore, the addition of color information on top of the shape information (from grayscale input) significantly jeopardized all DCNNs: the weakest impairment was found in VGG13, but the largest impairments were found in deeper ANNs (MobileNet and EfficientNet).

**Table 1:** The point of subjective equality (PSE) statistics for color and grayscale inputs with human data as the baseline in Experiment Part 1.

			**PSE**	***t*** **_(29)_**	***p***
Color Input	AlexNet		0.213	–12.709	<0.001
	VGG11		0.291	–6.045	<0.001
	VGG13		0.325	–3.192	0.047
	VGG16		0.210	–12.905	<0.001
	DenseNet		0.338	–2.038	0.711
	MobileNet		0.312	–4.287	0.003
	EfficientNet		0.213	–12.701	<0.001
Grayscale Input	AlexNet		0.361	–0.076	1.000
	VGG11		0.366	0.280	1.000
	VGG13		0.351	–0.993	1.000
	VGG16		0.397	2.971	0.083
	DenseNet		0.223	–11.826	<0.001
	MobileNet		0.431	–5.858	<0.001
	EfficientNet		0.215	–12.540	<0.001

The *p*-values are Bonferroni corrected.

**Figure 2:**
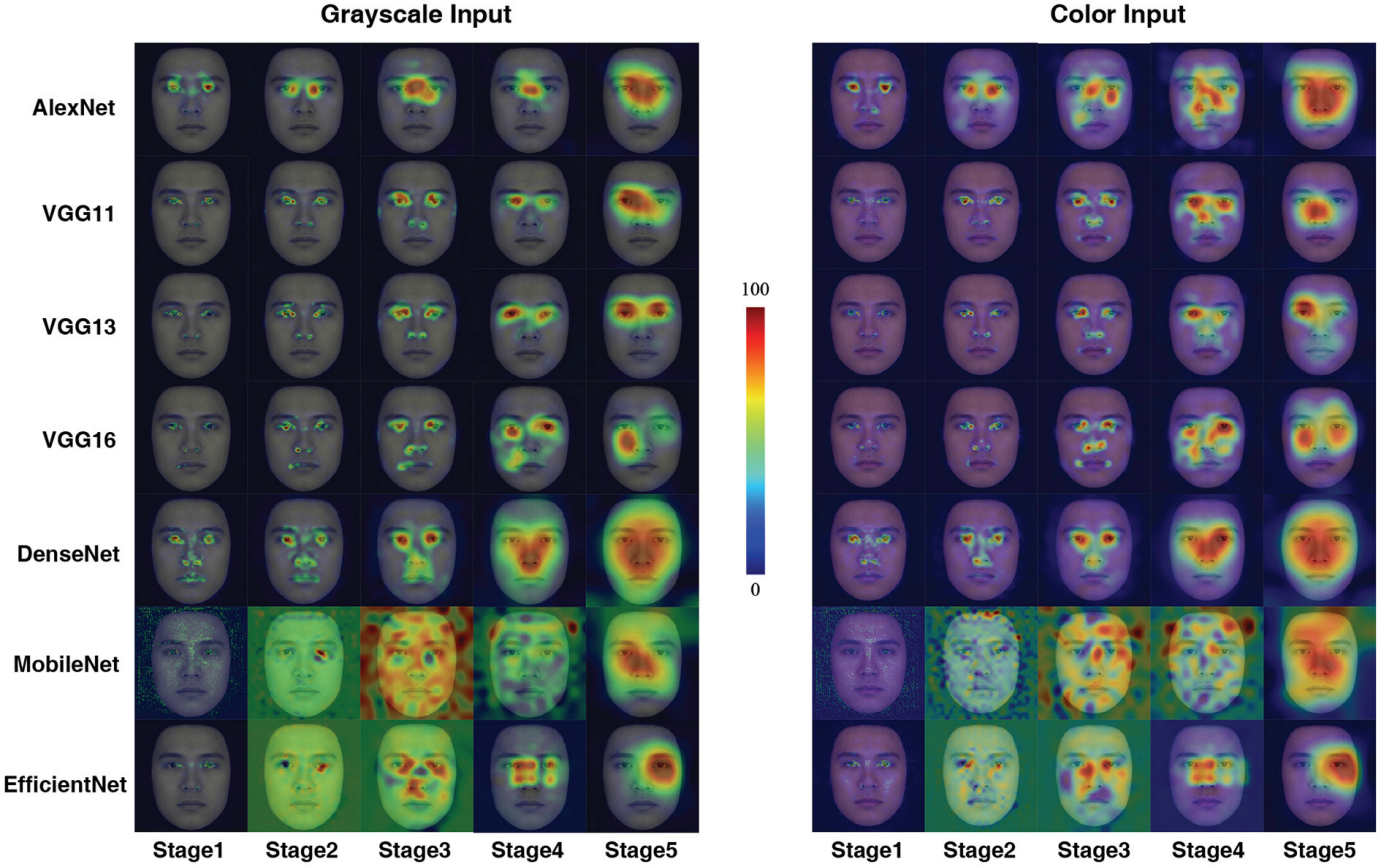
Activation maps for the seven models in grayscale and color conditions in Experiment Part 1. A sample image is used here for illustration purposes (The activation map shown below for Experiment 1 applies the same selection.). The term “stage” means the one generated after the final max-pooling activation of each stage. We know that VGG is a network composed of blocks, so each stage represents the stage at the end of each VGG block operation. For the stages where no max-pooling layer exists in the AlexNet section (stage3, stage4) the corresponding ReLu layer (layers 8, 10) is used instead. For DenseNet-201, stage 1 corresponds to the first max pooling layer in the network, stages 2–4 correspond to the three transition layers in the network, and stage 5 is the final Batch Normalization layer. For MobileNetV3-Small, stage 1 corresponds to the first convolution block, stages 2–4 to the 4th, 8th and 12th inversed residual block, and stage 5 to the last convolution block. For EfficientNetV2-S, stages 1–2 correspond to the 2nd and 10th Fused-MBConv blocks, and stages 3–5 correspond to the 6th, 15th, and 30th MBConv blocks.

To better understand what visual information the DCNNs used for processing, we used the CAM to interpret the “attention” of the DCNNs. As shown in Figure 2, all DCNNs extracted the eye region for face processing. However, it is clear that the AlexNet, at both input conditions, extracted information from a large region around the center of the eyes. Also, the CAM results suggested that VGG11, VGG16, and MobileNet shifted the attentional regions between two kinds of input conditions, suggesting that they extracted different visual inputs when the inputs were with or without color information. For the VGG13, DenseNet, and EfficientNet, the attentional shift was comparatively small among all seven DCNNs. The VGG13 concentrate on the eye region; the EffcientNet concentrated on the right eye region; the DenseNet concentrated on a larger region involving both eyes and the nose. Note that, the VGG13 was somewhat biased to the left eye at the color input condition. This leftward bias resembled the hemisphere lateralization found in human face perception (the face processing circuits were located in the right hemisphere, and thus humans rely more on the left part of the face for face processing Burt and Perrett, 1997; Megreya and Havard, 2011; Galmar et al., 2014; Tso et al., 2014).

#### 3.1.2. Brief discussion of experiment part 1

In the first part of the experiment, the results from analyzing the psychometric functions suggested that AlexNet, VGG11, and VGG13 would perform in a human-like fashion when dealing with only the shape information of a face (i.e., the grayscaled version of faces). However, the addition of color information significantly biased the DCNNs. Interstingly, the deeper DCNNs performed poorly with shape information and with additional color information. On the other hand, huge performance improvement with the additional color information found in DenseNet is staggeringly different from other DCNNs, suggesting its unique architecture. Thus, the results together suggested that the DCNNs are with computational mechanisms that are similar but different from humans.

The CAM method further illustrated that the attentional shifts of the DCNNs might explain the degeneration of performance. Although both VGG13 and EfficientNet attend to the eye region, the VGG13 focuses on the left eye (human-like), while the EfficientNet focuses on the right eye (opposite to human). It is reasonable to assume the human-like results of VGG13 can be explained by its information extraction methods. On the contrary, even though the EfficientNet is deeper and more advanced, its bias in information processing leads to a huge deviation from human performance. Interestingly, the CAM method showed that from Stage 2 to 4, the MobileNet has a complicated attention mapping. This pattern coincides with the distinguished convolution computation of MobileNet. Thus, this finding serves as an interesting piece of evidence supporting the utility of CAM method in unveiling the computational details of ANNs.

### 3.2. Experiment part 2: Testing DCNNs with essential facial features invisible

In the first part of the experiment, we found that the DCNNs underperformed when dealing with color inputs. The reason can be unveiled using CAM method (the attentional foci analysis). Following these findings, we decided to further validate what input information the DCNNs utilize when performing the ethnicity categorization task. To do so, we masked the essential regions of the faces. Inspired by the backward correlation method, it is clear that if a region of input is not used by a neural network for processing, then masking this region would hardly affect the performance of this network.

An increasing number of studies using eye-tracking analysis have supported the existence of certain patterns in human fixation with face information. In a recent study (Arizpe et al., 2017), researchers analyzed the spatial patterns of eye movements in normal healthy people and found four natural clusters. The clusters were more evenly distributed across the eyes, the bridge of the nose, and across the nose, philtrum, and upper lip. In the cross-cultural study of ethnicity recognition (Blais et al., 2008; Miellet et al., 2013), a combination of Western and Eastern gaze tendencies also revealed that human attention to ethnic distinctions is also focused on the position of the eyes and nose. The existence of such a specific pattern is also supported by the results of ethnicity classification studies based on different areas of interest (AOI) categories for different angles (Brielmann et al., 2014) and for children and adults (Hu et al., 2014). Therefore, if a DCNN is indeed human-like, then the occlusion of the essential facial feature for human would jeopardize the performance of that DCNN severely.

From this part of the experiment, we slightly altered the test inputs for the DCNNs. Using the OpenCV library, we generated a 162 × 63 black rectangle and masked the eyes, left eye, right eye, nose, and mouth on the original image of size 562 × 762. Thus, 6 sets of 21 face stream verification sets are generated simultaneously. In the subsequent CAM analysis, we scaled the image to a size of 224 × 224, and the corresponding occlusion was scaled equally.

#### 3.2.1. Results of experiment part 2

**Figure 3:**
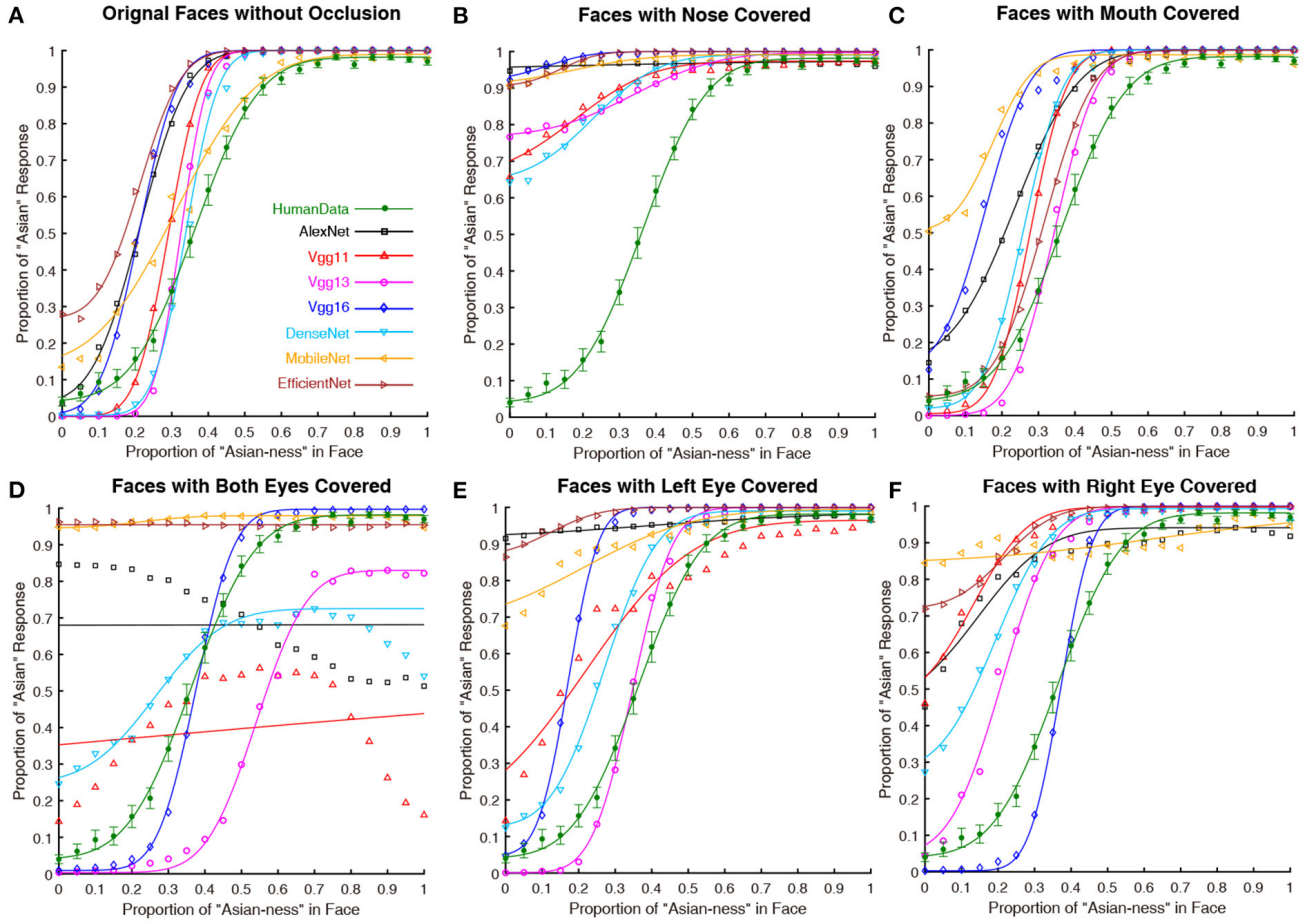
The psychometric functions of human participants and DCNNs under original and occluded input conditions in Experiment part 2. Green lines and symbols represent human participants, black lines and symbols represent AlexNet, red lines and symbols represent VGG11, magenta lines and symbols represent VGG13, and blue lines and symbols represent VGG16, azure lines and symbols represent DenseNet, yellow lines and symbols represent MobileNet and brown lines and symbols represent EfficientNet. The horizontal coordinates represent the change in “Asian-ness” of the input, and the vertical coordinates represent the transformed predicted probabilities corresponding to human subjects or DCNNs. Note that, the **(A)** is the same as Figure 1 (Left), we still present this subfigure for illustrative purpose only. **(B)** Faces with nose covered. **(C)** Faces with mouth covered. **(D)** Faces with both eyes covered. **(E)** Faces with left eye covered. **(F)** Faces with right eye covered.

The results of Experiment Part 2 were shown in Figure 3 and the results of the analysis over the PSE (point of subjective equality) were detailed in Table 2 (all of the *p* values were Bonferroni corrected). For the nose covered input condition (Figure 3B), all seven DCNNs have severally distorted psychometric functions. Thus, covering the nose region (an important hub for holistic processing found in human face perception) significantly biased the performance of all DCNNs tested here. At the mouth covered input condition (Figure 3C), the performances of most DCNNs (apart from MobileNet) tested here were similar to the performance in the original unprocessed input condition (with all facial features available; Figure 3A). For the both eyes covered input condition showed (Figure 3D), the results favored the notion that most DCNNs could not properly perform the face processing task. However, VGG16 [*t*_(29)_ = 1.095, *p* = 1.000] was similar to human participants based on the psychophysical function and statistical results of PSE. Comparing the left eye and right eye covered condition shown in Figures 3E,F, the performance of most DCNNs could not correctly perform the ethnicity judgment as they can when all visual features are available. Interestingly, the VGG16 [*t*_(29)_ = 0.781, *p* = 1.000] performed in a fashion that was closed to human participants in right eye covered condition and it is similar for VGG13 [*t*_(29)_ = –1.282, *p* = 1.000] in left eye condition.

**Table 2:** The point of subjective equality (PSE) statistics for different occlusion inputs with human data as the baseline in Experiment Part 2.

			**PSE**	***t*** **_(29)_**	***p***
Original	AlexNet		0.213	–12.709	<0.001
	VGG11		0.291	–6.045	<0.001
	VGG13		0.325	–3.192	0.074
	VGG16		0.210	–12.905	<0.001
	DenseNet		0.338	–2.038	1.000
	MobileNet		0.312	5.858	0.004
	EfficientNet		0.213	–12.540	<0.001
Both Eyes	AlexNet		▴	▴	▴
	VGG11		▴	▴	▴
	VGG13		0.541	15.197	<0.001
	VGG16		0.375	1.095	1.000
	DenseNet		▴	▴	▴
	MobileNet		▴	▴	▴
	EfficientNet		▴	▴	▴
Left Eye	AlexNet		▴	▴	▴
	VGG11		0.220	–12.081	<0.001
	VGG13		0.347	–1.282	1.000
	VGG16		0.171	–16.207	<0.001
	DenseNet		0.270	–7.845	<0.001
	MobileNet		▴	▴	▴
	EfficientNet		▴	▴	▴
Right Eye	AlexNet		▴	▴	▴
	VGG11		▴	▴	▴
	VGG13		0.212	–12.803	<0.001
	VGG16		0.372	0.781	1.000
	DenseNet		0.193	–14.382	<0.001
	MobileNet		▴	▴	▴
	EfficientNet		▴	▴	▴
Nose	AlexNet		▴	▴	▴
	VGG11		▴	▴	▴
	VGG13		▴	▴	▴
	VGG16		▴	▴	▴
	DenseNet		▴	▴	▴
	MobileNet		▴	▴	▴
	EfficientNet		▴	▴	▴
Mouth	AlexNet		0.237	–10.680	<0.001
	VGG11		0.278	–7.174	<0.001
	VGG13		0.343	–1.639	1.000
	VGG16		0.155	–17.625	<0.001
	DenseNet		0.260	–8.728	<0.001
	MobileNet		▴	▴	▴
	EfficientNet		0.315	–4.007	0.009

We did not report the PSE of condition with failure to fit or has a large bias. The *p*-values are Bonferroni corrected.

▴ Indicates that the data failed to fit a proper psychometric function or had a large bias. thus the results are not meaningful and had been discarded for analysis.

**Figure 4:**
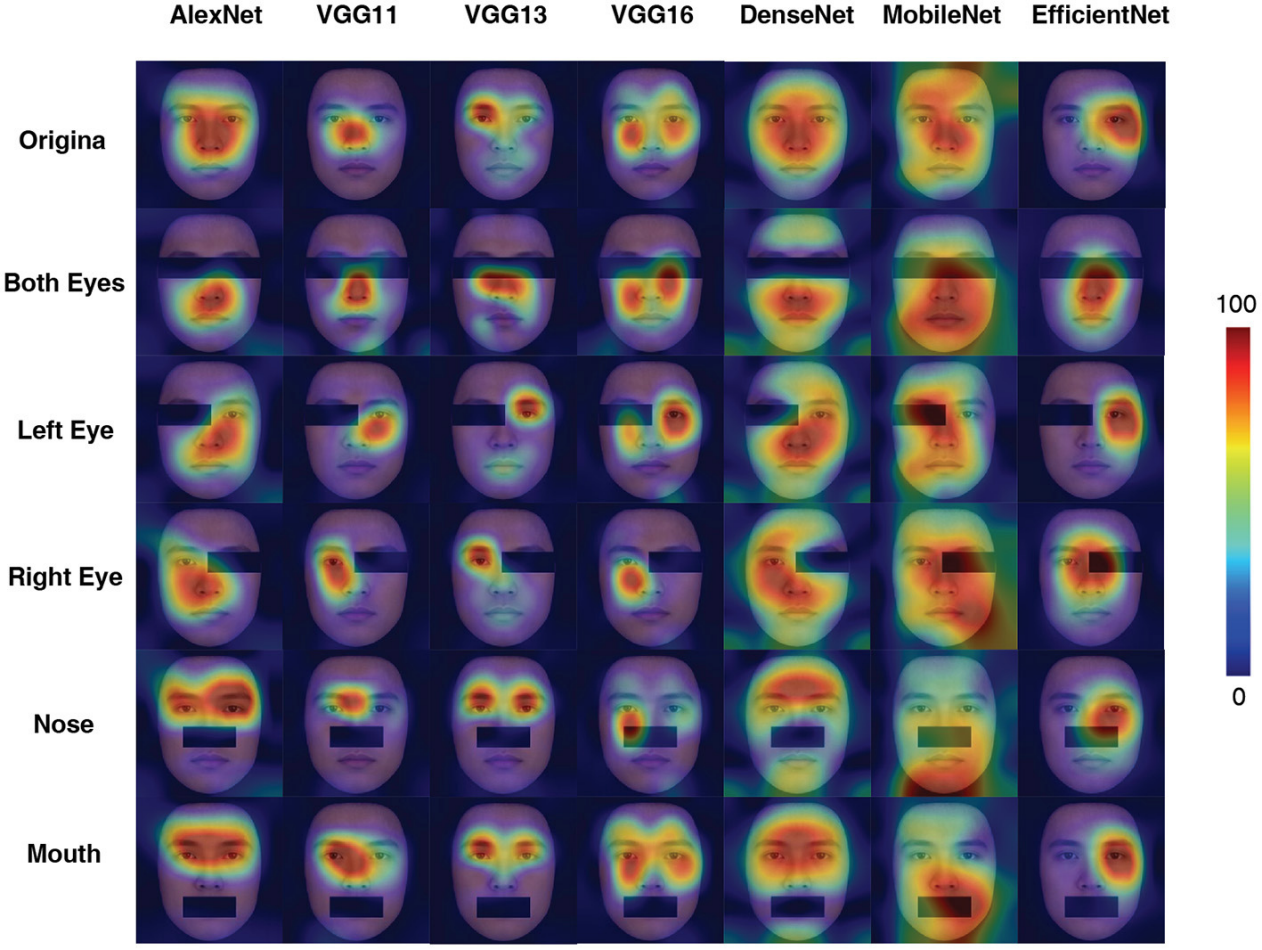
Activation maps for different networks under different occlusion conditions in Experiment Part 2. The figures were generated using the LayerCAM method. Note that, only the last “stage” is shown here.

Figure 4 illustrated the CAM outputs of the DCNNs (the last fully connected layer) processing the original as well as disrupted inputs at different conditions. Almost all DCNNs exhibited larger variations in the "attention" region compared to the original input in the masked both eyes condition, while the VGG16 has less variation. Comparing the results of the left eye and right eye masked conditions, there was a degree of asymmetry in the results, but this result did not seem to be significantly reflected. The attentional areas of all DCNNs were significantly affected in the control blocking nose condition. In contrast to this is the condition controlling for masking of the mouth, in which the effect on DCNNs was minimal (apart from MobileNet).

#### 3.2.2. Brief discussion of experiment part 2

Results of CAM and psychometric functions together suggested that the eye regions (especially for both eyes) are essential for DCNNs to perform the ethnicity categorization task: covering them significantly impaired the performance. Interestingly, the VGG16 seems to perform the task with little influence when covering the eyes (Figure 3D). Moreover, all DCNNs' performances were disrupted when the nose of the faces was covered, suggesting that the DCNNs do not solely rely on the eyes but are more likely to process the configural information of the faces (Blais et al., 2008). Previous researchers found that humans would concentrate on the nose region to better extract the configural information of a face for holistic processing (Van Belle et al., 2010; Linka et al., 2022). Therefore, from this point of view, all of the DCNNs grabbed an important computational mechanism as humans.

From the results in PSE *per se*, one may conclude that both VGG13 (best when the right eye covered condition) and VGG16 (best at both eye covered and left eye covered conditions) performed in human-like fashions at different conditions occluding eyes.

The asymmetry performances of the DCNNs of inputs with left (Figure 3E) and right (Figure 3F) eyes covered suggested that these DCNNs (especially for AlexNet) do not rely on inputs from both sides of face equally. It is well documented that human face perception is left-face biased, probably because the face processing brain circuits are in the right hemisphere (Kanwisher et al., 1997). Thus, a human-like DCNN should be more impaired when the left eye is covered than when the right eye is covered. The VGG16's performance was much better (and almost human-like) when the right eye is covered. Thus, it is reasonable to assume that the VGG16 captures the hemisphere lateralization found in the human neural system. However, considering the fact that VGG16 is not interrupted when both eyes are covered (Figure 3D), it is more likely that additional information from the right eye (when the left eye is covered; Figure 3E) severely impaired its performance.

### 3.3. Experiment part 3: Testing DCNNs with essential facial features visible

Based on the findings of Experiment Part 2, we expanded the experimental design by reversing the input impairment methods. Here, only the essential regions were *visible*. Studies in human face perception suggested that human utilizes holistic information (e.g., the second-order relationships of facial features) from faces to process faces (Maurer et al., 2002; Van Belle et al., 2010). Therefore, the essential facial features (e.g., eyes and nose) alone would not support face processing. On the other hand, if a DCNN process faces only using certain essential facial features, then it shall be able to perform the ethnicity categorization task with similar accuracy as the condition with all facial features visible.

In this part of the experiment, we carefully controlled the face inputs, leaving only the eye region, nose, and mouth of the faces visible to the DCNNs. In addition, we changed the size of the masks: (1) the same as Part 2, (2) 216 × 84 for the larger boxes, and (3) 90 × 35 for the smaller boxes.

#### 3.3.1. Results of experiment part 3

**Figure 5:**
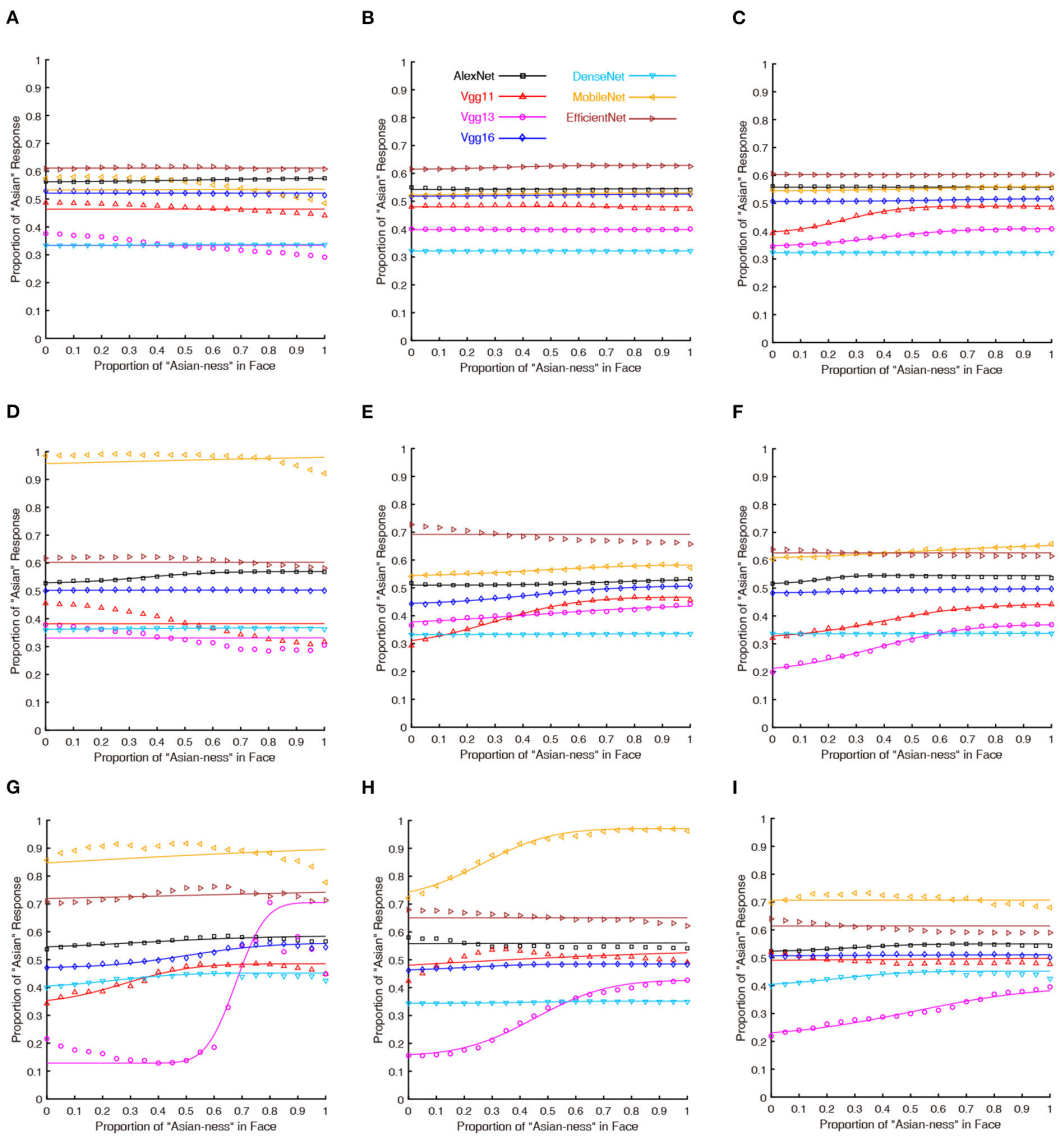
The psychometric functions of DCNNs under the conditions that only essential facial features are visible. Black lines and symbols represent AlexNet, red lines and symbols represent VGG11, magenta lines and symbols represent VGG13, and blue lines and symbols represent VGG16, azure lines and symbols represent DenseNet, yellow lines and symbols represent MobileNet and brown lines and symbols represent EfficientNet. The horizontal coordinates represent the change in “Asian-ness” of the input, and the vertical coordinates represent the transformed predicted probabilities corresponding to DCNNs. **(A)** Faces with Only eyes visible (small obstacle box). **(B)** Faces with only mouth visible (small obstacle box). **(C)** Faces with only nose visible (small obstacle box). **(D)** Faces with only eyes visible (obstacle box). **(E)** Faces with only mouth visible (obstacle box). **(F)** Faces with only nose visible (obstacle box). **(G)** Faces with only eyes visible (large obstacle box). **(H)** Faces with only mouth visible (large obstacle box). **(I)** Faces with only nose visible (large obstacle box).

The psychometric functions were illustrated in Figure 5. Almost every curve should be considered as disrupted, thus we did not further analyze the PSE. In other words, the DCNNs were no longer sensitive to the task when only the essential facial features are visible.

**Figure 6:**
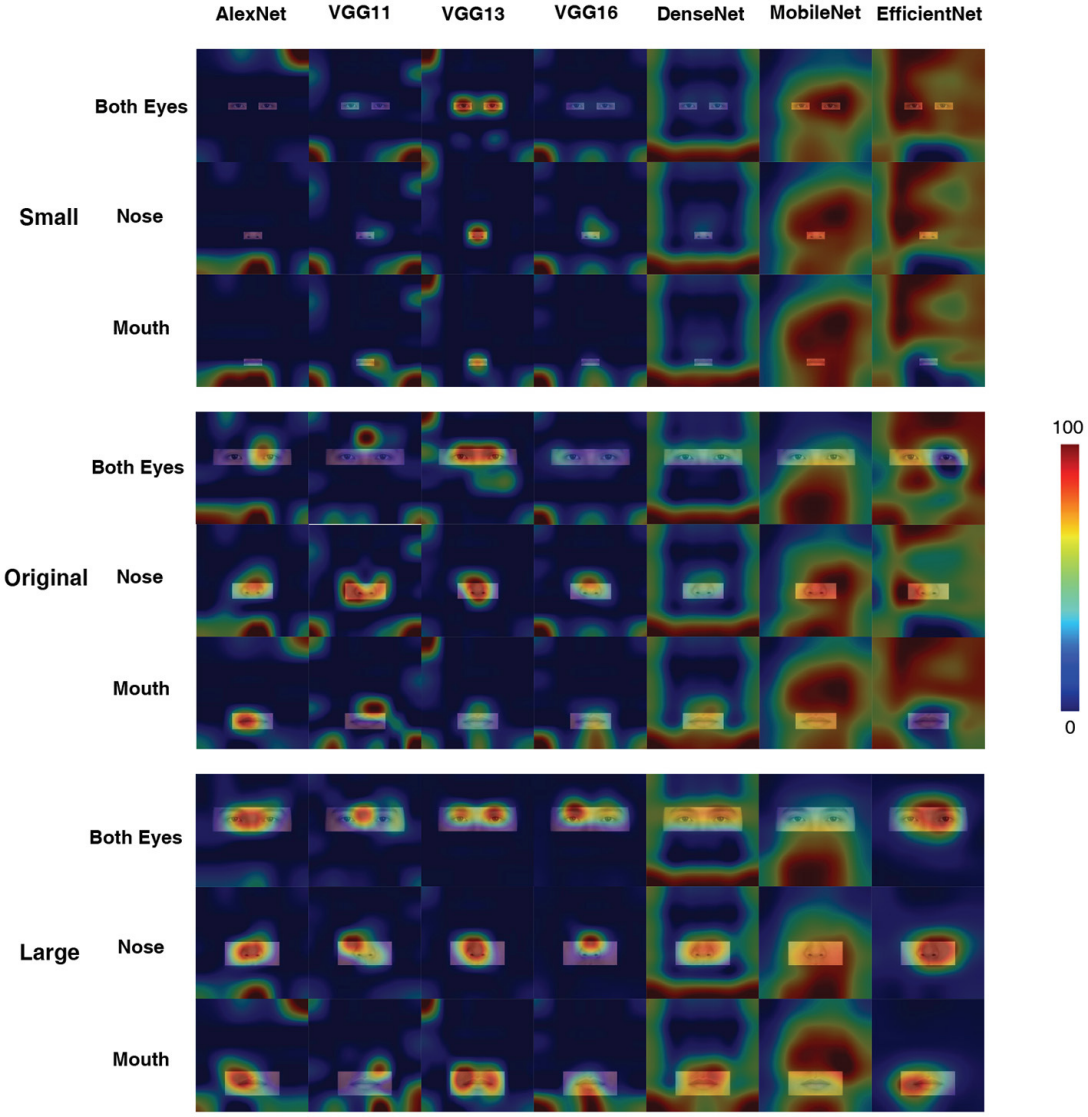
Activation maps for different networks in the case of three sizes of the display area, with only the eyes, nose, and mouth displayed. The illustrated figures were generated with the LayerCAM method. Note that, only the last “stage” was shown here.

Figure 6 showed the CAM output for the last layer of each DCNN. In most cases, the DCNNs concentrated on the visible areas of the face. However, the AlexNet, VGG11, VGG16, and DenseNet focused on the edge of the face, where no visual inputs were available (e.g., the AlexNet at Original Input condition, with Nose visible). Interestingly, the MobileNet focused on random locations at all sizes of display area; and the EfficientNet did so at most cases. Comparatively, the VGG13 stuck with the available visual inputs at all conditions.

#### 3.3.2. Brief discussion of experiment part 3

All of the DCNNs tested in this study failed to categorize the ethnicity of a target face with only the essential facial features available, regardless of the size of the mask. It is reasonable to conclude that the DCNNs do not rely on a single facial feature to process facial ethnicity. Consequently, the DCNNs do rely on holistic information from the face to perceive them.

## 4. General discussion

In this study, we explored the impact of various kinds of lossy inputs on the ethnicity categorization task performance of seven Deep Convolutional Neural Networks (DCNNs; AlexNet, VGG11, VGG13, VGG16, DenseNet, MobileNet, and EfficientNet) and examined their computational mechanisms using human performance as a benchmark. Three parts of experiments allowed us to explore the cognitive similarity between DCNNs and humans in terms of psychophysics function and the extraction of visual information. In the first part of the experiment, we tested DCNNs with grayscale and color image inputs. The results suggested that the AlexNet, VGG11, and VGG13 performed in a human-like fashion with grayscale inputs; however, the addition of color information significantly jeopardized the performance of most of the DCNNs apart from DenseNet, which performed better with color input. The results from CAM methods further unveiled that the DCNNs extracted different visual information from the faces, and thus confirmed that different DCNNs utilized different visual inputs to perform the same computational task. In the second part of the experiment, we adapted the reverse correlation method to unveil the information extraction pattern of the DCNNs the by testing the DCNNs with essential facial features occluded (disrupted inputs). Out of all seven DCNNs that were tested, the performance of VGG13 resembled human participants most closely, followed by the DenseNet. Based on the findings of Experiment Part 2, we only presented these facial features to the DCNNs in Experiment Part 3 and found that the DCNNs may need holistic information for face perception. Three parts of the experiment together verified the interference of lossy image inputs to the neural network by comparing psychophysical methods with State-of-The-Art techniques in visual artificial neural networks. In general, we examined the processing mechanism of DCNNs in light of previous findings in human face perception and found that VGG13 might be the most human-like DCNN in this task. These results together extended our understanding of DCNN and offered a new way to unveil the processing details of neural networks.

In the first part of the experiment, by comparing the performance of seven DCNNs under the input of grayscale and color conditions, we found that the results of the two produced large differences. In previous studies (Torres et al., 1999; Yip and Sinha, 2002; Choi et al., 2009), it has been generally accepted that color can transmit more useful information, but our experimental results indicate that DCNN can learn shape information about facial features well, and color information becomes a distracting factor, and here DCNN is not similar to human performance. Thus, future researchers in computer vision should be cautious when training and using different ANNs with or without color information.

Eyes, nose, and mouth regions, as well as the second order relationship among them (Maurer et al., 2002), are vital for humans to perceive a face (Blais et al., 2008; Miellet et al., 2013; Brielmann et al., 2014; Hu et al., 2014; Arizpe et al., 2017). Some researchers in face perception even argued that the ability to from the holistic representation is a hallmark for expertise in face processing (Maurer et al., 2002; Van Belle et al., 2010; Linka et al., 2022). According to the effects of the activation plots shown in Figure 2, the “perceptually” similar DCNNs extracted different parts of information from faces for the ethnicity categorization task. Thus, we employed the reverse correlation method (Song et al., 2021) to examine the DCNNs. In Experiment Part 2, we occluded these key facial information, and input these disrupted visual information to the DCNNs. In previous studies such as Itier et al. (2007), researchers found that the eye region is the most important region for human face recognition in visual information processing. However, both CAM and psychophysical results suggested that the VGG16 does not seem to be interested in the eyes. On the contrary, the VGG13 performed much better under all conditions. The fact that all networks fail to properly perform when the nose was obscured suggests that the area near the nose is also critical for the network, which is in line with the recent study (Linka et al., 2022). on humans, for whom positions close to the theoretical optimum point for face identification to be between the eyes and the nose.

In Experiment Part 3, we presented the DCNNs with only important facial features visible. The performance of DCNNs clearly indicated that they could not rely on these important face features alone for the face ethnicity classification task. It is reasonable to assume that the DCNNs need information about the structure of the face or the whole face to perform the correct face processing task, which is close to human visual processing (Maurer et al., 2002). The CAM results indicated that, except for VGG13, the network seems to be focusing on some irrelevant non-face regions (on the edge of the input images). Thus, the DCNNs (regardless of the “deep”-ness) still suffered from over-fitting issues. Here the results hinted that the DCNNs could hardly perceive the ethnicity of a face with only facial features, suggesting that they (at least partly) require the global information of a face for this high-level vision task. Although these DCNNs performed poorly in this part of the experiment, they do share similar processing characteristics to humans.

Among all seven DCNNs tested here, the results favored the conclusion that the VGG13 is the most human-like and the best ANN for the ethnicity categorization task. AlexNet had the worst performance among all DCNNs tested here, not only in terms of its poor robustness at various disrupted input conditions (e.g., Figure 3), but also in terms of its imprecise information extraction found in the CAM results. This finding is reasonable, as AlexNet is not a complex model, and its performance or "brain-like" properties are weaker than VGG in many studies (Song et al., 2021; Nicholson and Prinz, 2022; Zhou et al., 2022). VGG11 had a similar behavioral performance as AlexNet, but its information extraction was more human-like. The VGG13 achieved the most human-like performance across all experimental conditions. The VGG13 was able to accurately capture the key information in the input compared to other networks (Figure 6). As shown in Figure 5. Interestingly, the VGG13 also favored the left part of face like humans do. VGG16 also seems to reflect the characteristics of VGG13, but in general, it failed to achieve a robust result. For example, in the result of Experiment part 2 (in Figures 3, 4), the occlusion of the eye area did not interfere much with VGG16, indicating that it did not use eye regions for face processing, which is deviated from human data (Adolphs et al., 2005). The DenseNet can only perform human-like with the additional color information but utterly failed with only the shape information. The MobileNet as well as EfficientNet can hard extract the most important facial features and performed poorly. Why do deeper networks perform worse in this task than the VGG13? In a recent theoretical study (Sun et al., 2016) of the depth of neural networks, it is shown that as the network deepens, the Rademacher Average (RA; a measurement of complexity) increases accordingly, which has some negative effects on the network. From the information acquisition point of view, deeper networks may be overfitting in learning and learning something unimportant, so the deeper networks do not seem to be able to perform simple tasks on par with computationally less sophisticated networks.

The data from Experiment Part 2 suggested that the well-performed (human-like) VGG13 favored the right eye more than the left eye for the task ; while the relatively under-performed EfficientNet extracted mainly on the right eye. However, the VGG16, which does not need visual inputs from eyes, performed much better with left eye input than right eye input. It has been well established that people's ability to face processing is better when faces are presented in the left visual field, a phenomenon known as left visual field superiority (LVP superiority, De Renzi et al., 1968; Gazzaniga, 2000; Kanwisher and Yovel, 2006; Yovel et al., 2008). As visual information from each side of the visual field is sent to the contralateral hemisphere, convergent evidence from neuroimaging has indicated that the VLP superiority reflects the right hemispheric dominance for face perception (Kanwisher and Yovel, 2006; Meng et al., 2012). Specifically, the brain activation pattern in the left fusiform gyrus (known as Fusiform Face Area) is specially related to face processing (Kanwisher and Yovel, 2006; Meng et al., 2012). The VGG13 performed much better and human-like than VGG16; while they have opposite visual field superiority. It is worth noting that an ANN does not necessarily follow all specific lateralization characteristics of human neural system.

According to Marr's three-stage theory of computation (Marr, 1982/2010), although artificial neural networks are very different from biological neural networks at the level of hardware implementation, they may be consistent at the level of computational theory and even algorithms. The visual system is essentially an information processing system, and this system theory is similar to biological or machine neural networks, that is, there are similarities between biological and machine information acquisition levels from the visual perspective. This study (Zhang et al., 2018) demonstrates that a good feature can perform equally well in humans with different models, a result that supports this theory. which is the theoretical basis for our study. If we assume that a neural network is also an information processing system, then understanding its selectivity in processing information is an important task. In this study (Adolphs et al., 2005), it was shown that for humans, problems in the allocation of attention affect the processing and perception of faces, and combined with Marr's theory of vision, a well-performing neural network should be more similar to humans in terms of information acquisition. Thus, the information extraction, a good indicator of functional mechanism, is the most important evaluation perspective.

The interpretability of neural networks is always the focus of researchers in computer sciences. The current experiment endeavored to explore the interpretability of neural networks from the perspective of psychophysics methods. Apart from that, we also used the reverse correlation method with the findings in human perception in mind: we compared the information extraction of DCNNs with human face viewing patterns. To do so, we employed the State-of-The-Art visualization method CAM, a critically acclaimed method for visualizing and interpreting neural networks. Recently, a new passive attention technique seems to be able to visualize neural networks in a way that is closer to human understanding relative to CAM (Langlois et al., 2021), and future researchers should consider using the advanced version of this technique to further analyze the results. On the other hand, future researchers should also extend the present paradigm to neural networks trained with other face processing tasks and further validate this approach.

In summary, this study endeavored to examine the computational details of seven DCNNs (AlexNet, VGG11, VGG13, VGG16, DenseNet, MobileNet, and EfficientNet) using different behavioral methods by presenting different kinds of disrupted inputs to the DCNNs and tested the performance of the DCNNs with human benchmarks. The results suggested that: (1) these DCNNs have similar but different behavioral performance (psychometric function) as well as information acquisition patterns (CAM outputs) as humans; (2) different image disruption methods affected DCNNs' performance differently, and these NNs have different computational mechanisms and utilize different visual inputs for the same computational task; and (3) the VGG13 outperformed other DCNNs, the more complex networks do not necessarily perform better on simple tasks. In general, we examined the processing mechanism of DCNNs in light of previous findings in human face perception and found that VGG13 might be the most human-like DCNN in this task. These results together extended our understanding of DCNNs and offered a new way to unveil the processing details of neural networks.

## Data availability statement

The codes generated for this study can be found in the Open Science Framework (https://osf.io/9tpsd/).

## Ethics statement

The studies involving human participants were reviewed and approved by the Ethics Committee of Soochow University. The patients/participants provided their written informed consent to participate in this study.

## Author contributions

Y-FL data analysis, investigation, and writing suggestion. HY conceptualization, data analysis, funding and resources acquisition, and writing draft and revision. Both authors contributed to the article and approved the submitted version.

## Funding

HY is supported by the National Natural Science Foundation of China (32200850), the Natural Science Foundation of Jiangsu Province (BK20200867), and the Entrepreneurship and Innovation Plan of Jiangsu Province.

## Conflict of interest

The authors declare that the research was conducted in the absence of any commercial or financial relationships that could be construed as a potential conflict of interest.

## Publisher's note

All claims expressed in this article are solely those of the authors and do not necessarily represent those of their affiliated organizations, or those of the publisher, the editors and the reviewers. Any product that may be evaluated in this article, or claim that may be made by its manufacturer, is not guaranteed or endorsed by the publisher.
